# Lnc-PKD2-2-3/miR-328/GPAM ceRNA Network Induces Cholangiocarcinoma Proliferation, Invasion and 5-FU Chemoresistance

**DOI:** 10.3389/fonc.2022.871281

**Published:** 2022-07-29

**Authors:** Lei Zhang, Donglai Ma, Fujun Li, Gongcai Qiu, Dongsheng Sun, Zhaolin Zeng

**Affiliations:** Department of General Surgery, The 2nd Affiliated Hospital of Harbin Medical University, Harbin, China

**Keywords:** cholangiocarcinoma, lnc-PKD2-2-3, miR-328, GPAM, pathogenesis and chemosensitivity

## Abstract

**Purpose:**

Our previous study observed that long non-coding RNA PKD2-2-3 (lnc-PKD2-2-3) is related to advanced tumor features and worse prognosis in cholangiocarcinoma (CCA). Then, this study aimed to further explore the linkage between lnc-PKD2-2-3, miR-328, and GPAM, as well as their effects on regulating CCA viability, mobility, and chemosensitivity.

**Methods:**

Lnc-PKD2-2-3, miR-328, and GPAM expression in 30 pairs of CCA tumor and adjacent tissues, as well as in CCA cell lines, were determined. Two CCA cell lines (HuCCT1 and TFK1) were transfected by lnc-PKD2-2-3 overexpression plasmid, lnc-PKD2-2-3 siRNA, miR-328 inhibitor, and GPAM siRNA alone or in combination, followed by cell proliferation, apoptosis, invasion, and 5-FU chemosensitivity detection. Besides, xenograft mice were established for validation.

**Results:**

Lnc-PKD2-2-3 and GPAM were higher, whereas miR-328 was lower in CCA tissues versus adjacent tissues and also in CCA cell lines versus control cells; meanwhile, they were correlated with each other (all *P <*0.05). Lnc-PKD2-2-3 knockdown decreased CCA cell proliferation, invasion, and increased apoptosis (all *P <*0.05), but lnc-PKD2-2-3 overexpression exhibited the opposite and weaker effect. MiR-328 knockdown induced CCA cell proliferation and invasion and also attenuated the effect of lnc-PKD2-2-3-knockdown in these functions (all *P <*0.05). Subsequently, GPAM knockdown reduced CCA cell proliferation and invasion and also weakened the effect of miR-328-knockdown in these functions (all *P <*0.05). Additionally, lnc-PKD2-2-3 positively regulated GPAM while negatively regulating miR-328. MiR-328 negatively modified GPAM in CCA cells. Luciferase gene reporter assays verified that lnc-PKD2-2-3 directly bound miR-328 and miR-328 directly bound GPAM. Finally, the lnc-PKD2-2-3/miR-328/GPAM network also regulated the 5-FU chemosensitivity of CCA cells. *In vivo* experiments further revealed that lnc-PKD2-2-3 overexpression promoted tumor volume and weight but repressed tumor apoptosis in xenograft mice; meanwhile, it increased GPAM expression but decreased miR-328 expression (all *P <*0.05). Conversely, lnc-PKD2-2-3 knockdown exhibited the opposite effects (all *P <*0.05).

**Conclusion:**

Lnc-PKD2-2-3/miR-328/GPAM ceRNA network promotes CCA proliferation, invasion, and 5-FU chemoresistance.

## Introduction

Cholangiocarcinoma (CCA), although a relatively low-prevalence malignancy of the hepatobiliary system, is one of the deadliest cancers in the world (1–3). CCA exhibits a high variation of incidence, which is low in Europe (0.3–3.4 per 100,000) and high in Asia (2.5–14.5 per 100,000) (3). Notably, CCA presents with strong heterogeneity, not only is due to its origination (intrahepatic, perihilar, or distal) but also results from the molecule differences forming the tumor (4–6). That makes the CCA hard to treat appropriately and it often faces an unpleasant prognosis (7). Nowadays, the prior therapy of CCA is still surgical resection, with or without neoadjuvant/adjuvant therapies. However, due to unobvious onset and covert early symptoms, most of the CCA cases lose chances for surgery. That makes the prognosis of CCA even worse (8–10). To improve the prognosis of CCA prognosis, researchers and physicians are focusing more and more focus on the deep pathogenesis originating from CCA, which could explore the potential treatment option for CCA.

Long non-coding RNA (lncRNA), as a newly discovered type of RNA in the current century with a length of more than 200 bp but less protein-coding ability, has been discovered to be closely involved in the pathogenesis of various cancers (11–14). In the aspect of CCA, several specific lncRNAs have been discovered to participate in CCA initiation or progression (15–18). For instance, lncRNA SNHG3 promotes CCA cell proliferation and migration *via* mediating the miR-3173-5p/ERG axis (15); lncRNA FAM66C induces CCA growth, mobility, and glycolysis by modifying miR-23b-3p/KCND2 *in vitro* and *in vivo* (16); and lncRNA HOTTIP elevates drug resistance of gemcitabine and cisplatin *via* sponging miR-637 in CCA (18). However, the engagement of a great number of lncRNAs in CCA is not dragged out, and comprehensive exploration is lacking.

Our previous study identified a total of 4,223 upregulated and 4,596 downregulated lncRNAs in CCA tissues versus adjacent non-tumor tissues *via* microarray assay, then verified a key lncRNA (lnc-PKD2-2-3) that not only correlated with advanced tumor features but also promoted stemness and drug resistance in CCA (19). Following that, we further analyzed potential lncRNA–miRNA–mRNA regulatory networks implicated in CCA pathogenesis and discovered that the lnc-PKD2-2-3/miR-328/GPAM network may be essential to CCA. This study first assessed the linkage of lnc-PKD2-2-3, miR-328, and GPAM with clinicopathological features in CCA patients and then evaluated their intercorrelation and effect on regulating CCA viability, mobility, and drug sensitivity.

## Methods

### Patients, Samples, and Detections

A total of 30 primary CCA patients who underwent tumor resection from February 2019 to July 2021 were consecutively enrolled in this study, and the detailed eligible criteria were shown in our previous study (19). Then, their tumor and adjacent tissues were acquired and proposed for RT-qPCR detection for the measurement of lnc-PKD2-2-3, miR-328, and GPAM. The protocol has already been approved by the Ethics Committee as presented in our previous study (19).

### Bioinformatic Analysis

The lncRNA and mRNA microarray data derived from our previous study was further analyzed here (19) to sort the potential lncRNA–miRNA–mRNA regulatory network implicated in CCA pathogenesis. In brief, the top 10 differentially expressed lncRNAs (DElncRNAs) (5 upregulated and 5 downregulated) were selected by the rank of log_2_FC (fold change). The correlated differentially expressed mRNAs (DEmRNAs) of 10 dysregulated DElncRNAs were screened by Pearson’s correlation coefficient (>0.9 or <−0.9). The miRNA prediction of DElncRNAs and DEmRNAs was completed by miRanda (http://www.microrna.org/). The ceRNA (lncRNA–miRNA–mRNA) network was displayed with the igraph in the R packages. Then, the lnc-PKD2-2-3/miR-328/GPAM network was identified and deeply investigated in this study.

### Cell Culture

Human CCA cell lines, namely, HuH28, HuCCT1, RBE, and TFK1, were purchased from RIKEN BioResource Research Center (Koyadai, Japan), and then human normal biliary epithelial cells (HIBEpiC) were purchased from Sciencell Research Laboratories (Carlsbad, USA). The 10% fetal bovine (FBS, Gibco, USA) containing RPMI 1640 Medium (Gibco, USA) or epithelial cell medium (Sciencell, USA) was applied to culture CCA cells or HIBEpiC. Cell culture was performed in a 95% air/5% CO_2_ environment at 37°C.

### Transfection

The lnc-PKD2-2-3 or negative control (NC) DNA fragment was cloned into the pEX-2 vector (Genepharma, China) to construct an overexpression plasmid. NC small interference RNA (siRNA), lnc-PKD2-2-3 siRNA, GPAM siRNA, NC inhibitor, and miR-328 inhibitor were synthesized by Shanghai GenePharma Co., Ltd. (Shanghai, China). In the presence of Lipofectamine™ 2000 Transfection Reagent (Invitrogen, USA), 0.8 μg overexpression plasmid, 0.5 pM siRNA, or 0.5 pM inhibitor was transfected into HuCCT1 or TFK1 cells alone or together. The normally cultured cells were used as a control.

### Cell Proliferation

At 0, 24, 48, and 72 hours (h) after transfection, the cell proliferation was analyzed by cell counting kit-8 (CCK-8) (Beyotime, China). Cells were seeded in a 96-well plate with the amount of 1 × 10^4^ in 10% FBS-containing RPMI-1640. Then 10 μl of CCK-8 reagent mixed with 100 μl of FBS-free RPMI-1640 was added and incubated with the cells at 37°C for 2 h. Finally, a microplate reader (BioTek, USA) was used to read optical density (OD) values under 450 nm.

### Cell Apoptosis

At 48 h after transfection, the TUNEL Apoptosis Assay Kit (Beyotime, China) was applied to assess cell apoptosis. In brief, first the cells were fixed at room temperature with 10% neutral formalin (Sangon, China) for 30 minutes (min). Secondly, the cells were incubated with TUNEL working solution in the dark at 37°C for 1 h. Thirdly, the images were captured using an inverted fluorescence microscope (Motic, China).

### Invasion

Transwell was conducted to measure invasion ability at 48 h after transfection. The transwell insert was coated with Matrigel Basement Membrane Matrix (BD, USA) at 37°C for 1 h. The cells (5 × 10^4^) in FBS-free RPMI-1640 were added to the insert, which was plated into a 24-well plate. The on-invasive cells were wiped out after being incubated at 37°C for 24 h, while the invasive cells were fixed and stained at room temperature. An inverted microscope (Motic, China) was used to take pictures.

### RT-qPCR

For clinical samples, the RT-qPCR was performed after sample acquirement; for cell samples, the RT-qPCR was performed 48 h after transfection. The RNA was extracted with Beyozol (Beyotime, China). The RNA with a concentration of 1 μg was transcribed by the RT reagent Kit (Takara, Japan). The thermal cycle of reverse transcription was as follows: 37°C for 15 min, 85°C for 5 seconds (s). The qPCR was completed by TB Green^®^ Fast qPCR Mix (Takara, Japan) and the following thermal cycles were conducted: 95°C for 30 s, 1 cycle; 95°C for 5 s, and 61°C for 15 s, 40 cycles. The results were calculated using the 2^−ΔΔCt^ method. The primer sequences (5’->3’) were as follows: lnc-PKD2-2-3, forward, AGGCTGATTCTGGAAGTTCTGAG, reverse, AGGAGATTCTGCTTCTGAGATGG; GPAM, forward, AGAATGAGAGCCTGTGGAGTGTA, reverse, TCTACCTTCATCAGCAGCATCAC; GAPDH, forward, GAGTCCACTGGCGTCTTCAC, reverse, ATCTTGAGGCTGTTGTCATACTTCT; miR-328, forward, GGGGGCAGGAGGGGC, reverse, GTCGTATCCAGTGCAGGGTCCGAGGTATTCGCACTGGATACGACCCCTGA; U6, forward, CTTCGGCAGCACATATACTA, reverse, AATATGGAACGCTTCACGAA.

### Western Blot

At 48 h after transfection, RIPA lysis buffer (Beyotime, China) was utilized to lyse the cells. The total protein was then quantified with a BCA kit (Sangon, China). The 20 μg of protein was separated by 4–20% precast gel (Willget, China), and then transferred to a nitrocellulose membrane (PALL, USA). The membrane was then blocked by 3% skim milk (Beyotime, China) at 37°C for 45 min, followed by incubation with GPAM antibody (1:100) (Invitrogen, USA) or GAPDH antibody (1:5,000) (Invitrogen, USA) at 4°C overnight. After being incubated with goat anti-rabbit antibody (1:2,000) (Beyotime, China) at 37°C for 90 min, the membrane was incubated with ECL substrate (Beyotime, China), and exposed with X-ray film (Kodak, USA). Quantification of protein bands was performed by ImageJ 1.8.0 (NIH, USA).

### Drug Sensitivity

Drug sensitivity was measured 48 h after transfection. The 4 × 10^4^ cells were seeded in a 96-well plate with 10% FBS-containing RPMI-1640. Then, 200 ng/ml of 5-fluorouracil (5-FU) (MCE, China) was cultured with cells for another 24, 48, and 72 h. For cell viability measurement, 10 μl CCK-8 reagent in 100 μl RPMI-1640 was added and cultured with cells for 2 h at 37°C. Finally, the OD value at 450 nm was measured by a microplate reader. The relative cell viability when setting the control group as the reference is calculated as follows: cell viability of each group/cell viability of the control group × 100%. Relative cell viability (5-FU/No treatment) when setting no treatment as a reference was also calculated as follows: cell viability under 5-FU/cell viability with no treatment × 100%.

### Luciferase Gene Reporter

The miR-328 mimic and NC mimic were designed and synthesized by Shanghai GenePharma Co., Ltd. (Shanghai, China). The lnc-PKD2-2-3 wild type (WT) plasmid, lnc-PKD2-2-3 mutant type (MT) plasmid, GPAM WT plasmid, and GPAM MT plasmid were constructed with the pGL6 vector (Beyotime, China). For the binding detection of lnc-PKD2-2-3 and miR-328, 0.8 μg of lnc-PKD2-2-3 WT/MT plasmid and 0.5 pM of miR-328/NC mimic were co-transfected into HuCCT1/TFK1/293T cells (ATCC, USA) and incubated for 48 h. Then, 293T cells were lysed and incubated with a Luciferase Reporter Gene Assay Kit (Beyotime, China). In the end, the luciferase activity was measured by a fluorescence microplate reader (BioTek, USA). For the binding detection of miR-328 and GPAM, 0.8 μg of GPAM WT/MT plasmid and 0.5 pM of miR-328/NC mimic were co-transfected into 293T cells. After 48 h, the cell lysis and luciferase activity detection were completed as mentioned above.

### *In Vivo* Experiments

The animal experimental procedures were conducted in accordance with the guidelines of our institution. The procedures were approved by the animal ethic committee of our institution. Nude mice were obtained from SLAC (Shanghai, China) and maintained in a 12 h light/dark, specific pathogen free condition. The HuCCT1 cells were infected with scramble, lnc-PKD2-2-3 overexpression, and lnc-PKD-2-3 knockdown lentivirus (Genepharma, China) to generate scramble, oeLnc, and shLnc cells, respectively. The scramble, oeLnc, and shLnc cells were inoculated subcutaneously into the dorsal flanks of mice (N = 6 for each group) with an amount of 2.5 × 10^6^. The tumor sizes were measured every week for 4 weeks and calculated using the formula: volume = length × width^2^/2. The mice were sacrificed at 4 weeks after cell implantation. The tumors were harvested and weighed. Each tumor was split into two, and stored in −80°C or embedded in paraffin. The paraffin embedded tumors were cut into 4 μm sections for hematoxylin–eosin (HE), TdT-mediated dUTP-biotin nick end labeling (TUNEL), and immunohistochemistry (IHC) staining, with the involvement of HE staining kit (Sangon, China), TUNEL staining kit (Beyotime, China), and GPAM antibody (1:40) (Invitrogen, USA). The frozen tumors were lysed for RT-qPCR and western blot.

### Statistical Analysis

In the aspect of clinical analysis, data were shown as count (%) or median (interquartile range (IQR)); comparison between two groups or among three groups was determined by Wilcoxon rank sum test or Kruskal–Wallis test, correlation between two variables was detected by spearman test. In terms of experimental analysis, data were shown as mean ± standard deviation; comparison among groups was determined by one-way ANOVA followed by Tukey’s or Dunnett’s multiple comparisons test. GraphPad Software 7.0 (GraphPad Int., USA) or SPSS software (IBM, USA) was adopted to complete data analysis. Statistical significance was defined as *P <*0.05.

## Results

### Dysregulated Expression of lnc-PKD2-2-3, miR-328, and GPAM in CCA Patients

**Figure 1:**
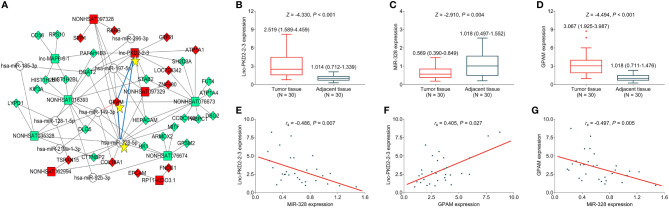
Measurement of lnc-PKD2-2-3, miR-328 and GPAM in CCA patients. Identification of lnc-PKD2-2-3/miR-328/GPAM network related to CCA pathology **(A)**. Comparison of lnc-PKD2-2-3 **(B)**, miR-328 **(C)**, and GPAM **(D)** between tumor and adjacent tissues. Correlation between lnc-PKD2-2-3 and miR-328 **(E)**, between lnc-PKD2-2-3 and GPAM **(F)**, and between miR-328 and GPAM **(G)**.

Further bioinformatic analysis using our previous data (19) suggests that the lnc-PKD2-2-3/miR-328/GPAM network might be closely involved in the CCA pathology (**Figure 1A**). By validation using RT-qPCR in 30 CCA patients, it was observed that lnc-PKD2-2-3 and GPAM were elevated, whereas miR-328 was reduced in CCA tumor tissues compared with adjacent tissues (all *P <*0.01) (**Figures 1B–D**). Furthermore, tumor lnc-PKD2-2-3 was negatively related to miR-328 but positively linked with GPAM in CCA (both *P <*0.05); meanwhile, tumor miR-328 negatively related to GPAM in CCA (*P <*0.01) (**Figures 1E–G**).

### Clinical Implication of lnc-PKD2-2-3, miR-328, and GPAM in CCA Patients

Tumor lnc-PKD2-2-3 positively correlated with poor differentiation and N stage (both *P <*0.05), but did not relate to other clinicopathological features in CCA patients (**Table 1**). Additionally, tumor miR-328 was linked with less advanced T, N, and TNM stages (all *P <*0.05). In the aspect of GPAM, it was positively associated with advanced T stage, N stage, and TNM stage (all *P <*0.05). No other linkage of lnc-PKD2-2-3, miR-328, or GPAM with clinicopathological features was observed.

**Table 1:** Correlation of lnc-PKD2-2-3, miR-328 and GPAM with clinical features of CCA patients.

Items	Patients, n (%)(N = 30)	Lnc-PKD2-2-3, median (IQR)	*P* value	MiR-328, median (IQR)	*P* value	GPAM, median (IQR)	*P* value
Age (years)			0.601		0.706		0.917
≤60	17 (56.7)	2.850 (1.684-4.551)		0.513 (0.334-0.867)		3.058 (2.031-4.068)	
>60	13 (43.3)	2.038 (1.548-3.677)		0.616 (0.416-0.808)		3.075 (1.727-4.876)	
Gender			0.836		0.092		0.097
Female	6 (20.0)	2.552 (1.049-6.923)		0.396 (0.292-0.615)		4.792 (2.065-6.661)	
Male	24 (80.0)	2.499 (1.650-4.123)		0.631 (0.432-0.893)		2.991 (1.682-3.767)	
Smoke			0.917		0.384		0.520
No	15 (50.0)	2.460 (1.630-4.736)		0.562 (0.424-0.896)		3.616 (2.100-3.905)	
Yes	15 (50.0)	2.537 (1.465-4.366)		0.576 (0.311-0.732)		2.923 (1.637-4.310)	
Drink			0.253		0.379		0.202
No	20 (66.7)	2.571 (1.731-4.644)		0.569 (0.402-0.732)		3.632 (1.887-4.932)	
Yes	10 (33.3)	2.350 (1.036-3.535)		0.736 (0.345-1.127)		2.685 (1.813-3.454)	
HBV infection			0.914		0.322		0.747
No	19 (63.3)	2.537 (1.630-4.736)		0.616 (0.424-0.883)		3.075 (2.100-3.784)	
Yes	11 (36.7)	2.500 (1.465-4.366)		0.488 (0.311-0.732)		3.058 (1.525-5.274)	
ECOG PS			0.220		0.078		0.107
0	19 (63.3)	2.296 (1.465-3.392)		0.645 (0.395-0.983)		2.832 (1.637-3.784)	
1 or 2	11 (36.7)	2.850 (2.460-4.736)		0.454 (0.236-0.680)		3.648 (2.538-5.968)	
Tumor site			0.530		0.620		0.825
Intrahepatic	8 (26.7)	1.918 (1.289-3.159)		0.520 (0.322-0.824)		3.395 (2.055-3.878)	
Perihilar	14 (46.6)	2.571 (1.647-5.176)		0.549 (0.390-0.772)		3.014 (1.590-5.976)	
Distal	8 (26.7)	2.730 (1.335-4.074)		0.647 (0.463-1.044)		2.991 (2.210-3.640)	
Differentiation			0.021		0.145		0.343
Well	6 (20.0)	1.501 (0.895-2.496)		0.782 (0.538-1.291)		2.954 (1.389-4.165)	
Moderate	13 (43.3)	2.537 (1.753-4.064)		0.562 (0.385-0.732)		3.616 (1.727-3.845)	
Poor	11 (36.7)	2.850 (2.296-5.588)		0.454 (0.236-0.983)		3.058 (2.538-5.968)	
T stage			0.061		0.040		0.025
T1 ~ T2	18 (60.0)	2.167 (1.323-3.109)		0.706 (0.475-0.886)		2.218 (1.590-3.843)	
T3 ~ T4	12 (40.0)	3.121 (2.496-4.741)		0.439 (0.241-0.573)		3.700 (2.957-4.932)	
N stage			0.006		0.016		0.021
N0	19 (63.3)	1.798 (1.175-2.610)		0.680 (0.488-0.983)		2.588 (1.637-3.714)	
N1 ~ N2	11 (36.7)	3.197 (2.604-4.736)		0.436 (0.236-0.576)		3.795 (3.058-5.968)	
TNM stage			0.055		0.010		0.020
I ~ II	17 (56.7)	2.038 (1.274-2.904)		0.732 (0.505-0.940)		2.336 (1.581-3.681)	
III ~ IV	13 (43.3)	3.079 (2.480-4.740)		0.436 (0.246-0.569)		3.784 (2.945-5.621)	

CCA, cholangiocarcinoma; Lnc, long non-coding; IQR, interquartile range; miR, microRNA; GPAM, glycerol-3-phosphate acyltransferase; HBV, hepatitis B virus; ECOG PS, Eastern Cooperative Oncology Group Performance Status.

### Aberrant Expression of lnc-PKD2-2-3, miR-328, and GPAM in CCA Cell Lines

**Figure 2:**
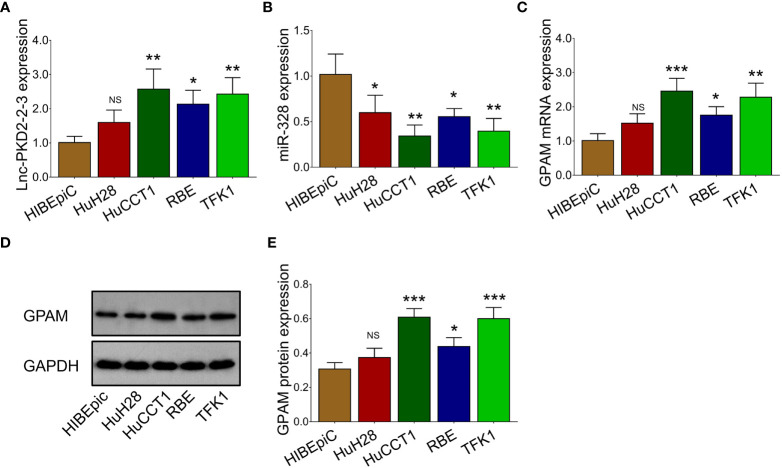
Measurement of lnc-PKD2-2-3, miR-328 and GPAM in CCA cell lines. Comparison of lnc-PKD2-2-3 **(A)**, miR-328 **(B)**, and GPAM **(C–E)** between CCA cell lines and control cell line. NS, not significant; *P <0.05; **, *P <*0.01; ***P <0.001.

The expression of lnc-PKD2-2-3, miR-328, and GPAM was further validated in CCA cell lines to uncover their potential engagement in CCA. It was observed that lnc-PKD2-2-3 was increased in multiple CCA cell lines compared to the control cell line (most *P <*0.05) (**Figure 2A**), while miR-328 was decreased in all CCA cell lines compared with the control cell line (all *P <*0.05) (**Figure 2B**). Additionally, GPAM was raised in multiple CCA cell lines compared to control cell line (most *P <*0.05) (**Figures 2C–E**).

### Lnc-PKD2-2-3 Regulated CCA Cell Proliferation, Apoptosis, and Invasion *via* Sponging miR-328

**Figure 3:**
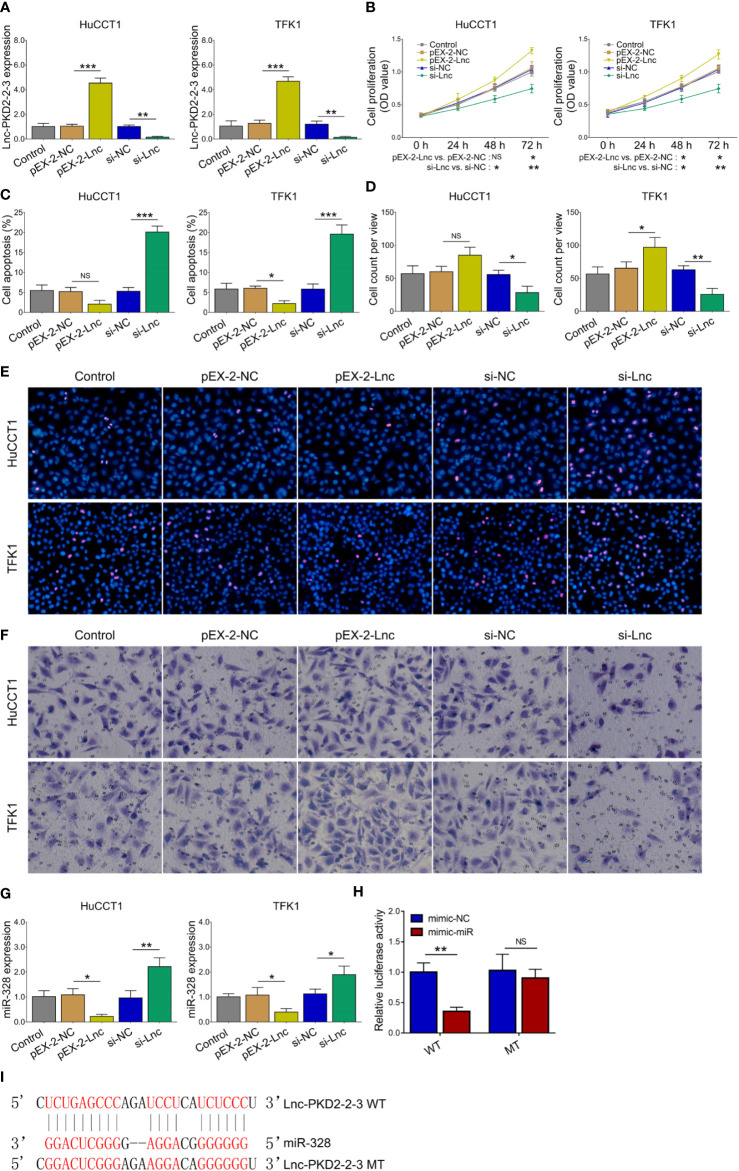
Effect of lnc-PKD2-2-3 on cell proliferation, apoptosis, invasion and miR-328 in CCA cell lines. Lnc-PKD2-2-3 expression **(A)**, cell proliferation **(B)**, cell apoptosis rate **(C)**, invasive cell count **(D)**, TUNEL image examples **(E)**, and Transwell image examples **(F)** among groups after transfection. MiR-328 expression among groups after transfection **(G)**, relative activity of luciferase gene reporter assay **(H)**, and bound/designed sequences of lnc-PKD2-2-3 and miR-328 **(I)**. NS, not significant; *P <0.05; **P <0.01; ***P <0.001.

The lnc-PKD2-2-3 was determined to be greatly modified after transfection in both HuCCT1 and TFK1 cells (all *P <*0.01), indicating that the transfection was successful (**Figure 3A**). Then, it was discovered that lnc-PKD2-2-3 overexpression promoted cell proliferation (reflected by OD value) (*P <*0.05), but less affected TUNEL-reflected cell apoptosis rate and Transwell-reflected invasion ability (both *P >*0.05) in HuCCT1 cells; notably, lnc-PKD2-2-3 knockdown reduced cell proliferation and invasion, while elevated cell apoptosis rate in HuCCT1 cells (all *P <*0.05) (**Figures 3B–F**). In the aspect of TFK1 cells, lnc-PKD2-2-3 overexpression enhanced cell proliferation and invasion, attenuated cell apoptosis (all *P <*0.05); whereas lnc-PKD2-2-3 knockdown exhibited an opposite effect (all *P <*0.05) (**Figures 3B–F**).

Additionally, lnc-PKD2-2-3 negatively regulated miR-328 in both HuCCT1 and TFK1 cells (all *P <*0.05) (**Figure 3G**). Subsequent luciferase gene reporter assays verified their direct binding *via* sequence in 293T cells (**Figures 3H,I**), HuCCT1 cells (**Supplementary Figure 1A**), and TFK1 cells (**Supplementary Figure 1B**).

**Figure 4:**
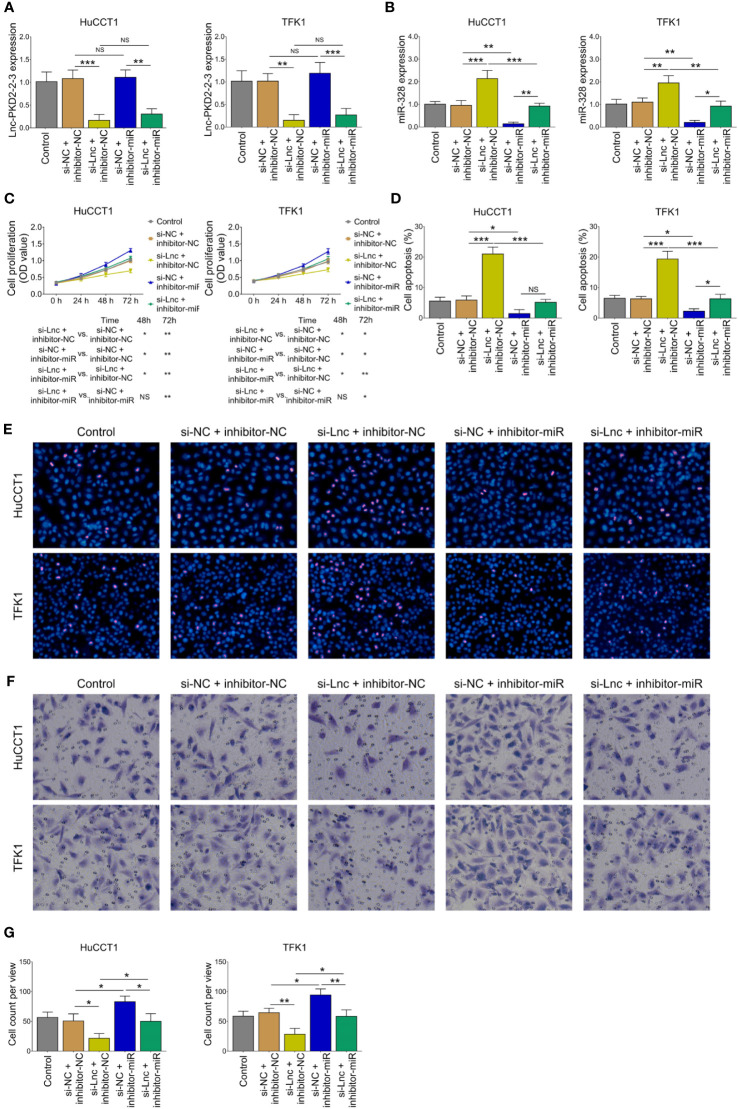
Effect of miR-328 knockdown on cell proliferation, apoptosis, and invasion in CCA cell lines. Lnc-PKD2-2-3 expression **(A)**, miR-328 expression **(B)**, cell proliferation **(C)**, cell apoptosis rate **(D)**, TUNEL image examples **(E)**, Transwell image examples **(F)**, and invasive cell count **(G)** among groups after transfection. NS, not significant; *P <0.05; **P <0.01; ***P <0.001.

The miR-328 inhibitor was then transfected into HuCCT1 and TFK1 cells as well. Lnc-PKD2-2-3 was determined to be largely decreased after transfection of lnc-PKD2-2-3 siRNA but not miR-328 inhibitor; meanwhile, miR-328 was determined to be greatly reduced after transfection of miR-328 inhibitor in both HuCCT1 and TFK1 cells (**Figures 4A, B**). Subsequently, cell proliferation at 48 h (*P <*0.05) and 72 h (*P <*0.01) was increased (**Figure 4C**), the cell apoptosis rate was decreased (*P <*0.05) (**Figures 4D, E**), and cell invasion was lowered (*P <*0.05) (**Figures 4F, G**), by miR-328 knockdown in HuCCT1 cells. Simultaneously, miR-328 knockdown exhibited a similar effect on the above functions in TFK1 cells as those in HuCCT1 cells (all *P <*0.05). It is worth noting that miR-328 knockdown attenuated the effect of lnc-PKD2-2-3 knockdown on cell proliferation at 48 and 72 h, cell apoptosis rate, and cell invasion in both HuCCT1 and TFK1 cells (all *P <*0.05) (**Figures 4C–G**).

### MiR-328 Regulated CCA Cell Malignant Behaviors *via* Targeting GPAM

**Figure 5:**
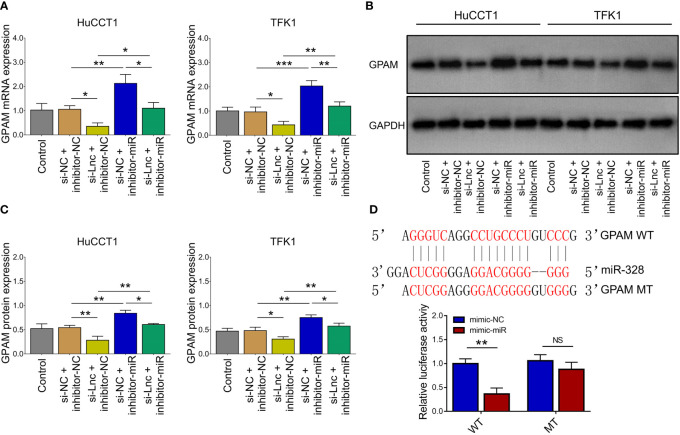
Effect of lnc-PKD2-2-3 knockdown and miR-328 knockdown on GPAM in CCA cell lines. GPAM expression among groups after transfection **(A–C)**. Relative activity of luciferase gene reporter assay and bound/designed sequences of miR-328 and GPAM **(D)**. NS, not significant; *P <0.05; **P <0.01; ***P <0.001.

Lnc-PKD2-2-3 knockdown decreased GPAM mRNA and protein expressions; while miR-328 knockdown increased GPAM mRNA and protein expressions, as well as weakened the regulatory effect of lnc-PKD2-2-3 knockdown on GPAM mRNA and protein expressions (all *P <*0.05) (**Figures 5A–C**). Followed luciferase gene reporter assay confirmed that miR-328 directly bound to GPAM *via* sequence in 293T cells (**Figure 5D**), HuCCT1 cells (**Supplementary Figure 1C**), and TFK1 cells (**Supplementary Figure 1D**), which was reflected by relative luciferase activity (all *P <*0.01). Combining the above data about the interaction among lnc-PKD2-2-3, miR-328, and GPAM, lnc-PKD2-2-3/miR-328/GPAM was suggested to be a ceRNA network.

**Figure 6:**
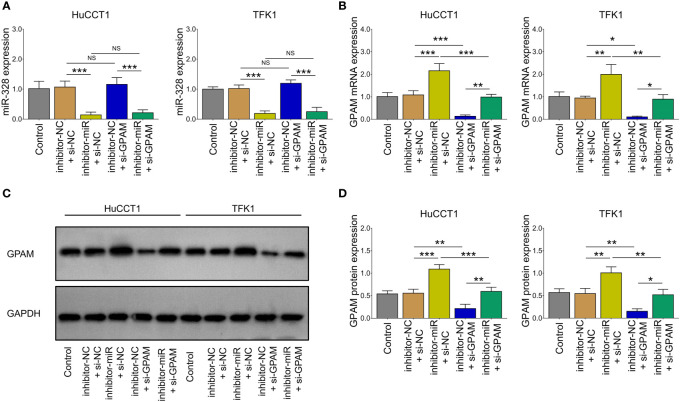
miR-328 and GPAM expressions after their siRNA/inhibitor transfection in CCA cell lines. miR-328 expression **(A)** and GPAM expression **(B–D)** among groups after transfection. NS, not significant; *P <0.05; **P <0.01; ***P <0.001.

**Figure 7:**
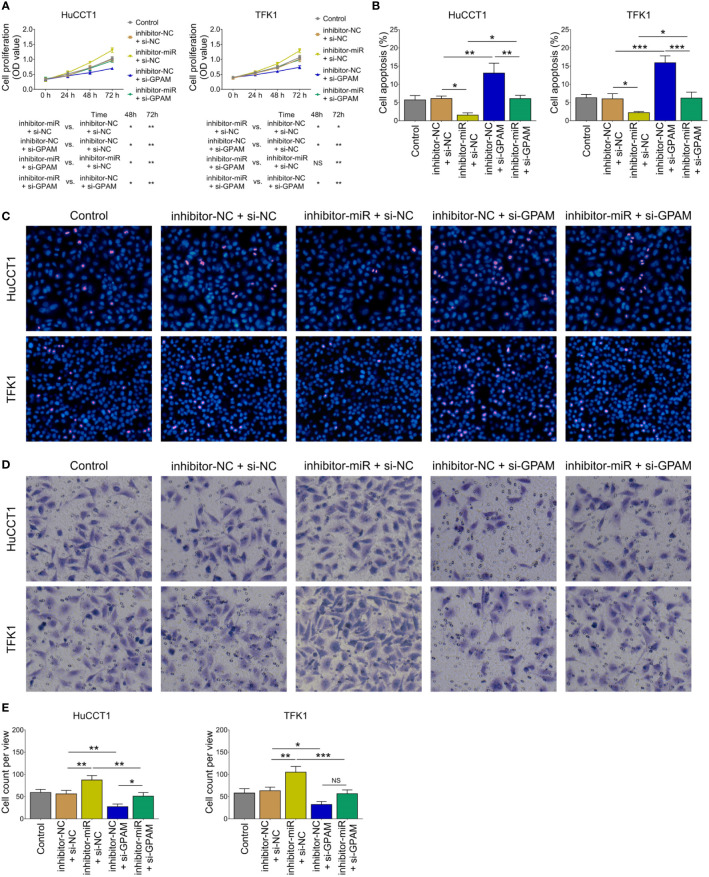
Effect of GPAM knockdown on cell proliferation, apoptosis, and invasion in CCA cell lines. Cell proliferation **(A)**, cell apoptosis rate **(B)**, TUNEL image examples **(C)**, Transwell image examples **(D)**, and invasive cell count **(E)** among groups after transfection. NS, not significant; *P <0.05; **P <0.01; ***P <0.001.

Inhibition of miR-328 was determined to be largely lowered after transfection of miR-328 inhibitor (*P <*0.001) but was not affected by GPAM siRNA (P >0.05) both in HuCCT1 and TFK1 cells (**Figure 6A**). GPAM protein and mRNA expression were increased by the miR-328 inhibitor (*P <*0.05) and greatly reduced after transfection of GPAM siRNA (*P <*0.05) (**Figures 6B–D**). Subsequently, cell proliferation at 48 h (*P <*0.05) and 72 h (*P <*0.01) was reduced (**Figure 7A**), the cell apoptosis rate was elevated (*P <*0.01) (**Figures 4B, C**), and cell invasion was repressed (*P <*0.01) (**Figures 7D, E**) by GPAM knockdown in HuCCT1 cells. Meanwhile, GPAM knockdown exhibited a similar effect on the above functions in TFK1 cells as those in HuCCT1 cells (all *P <*0.05). It is worth noting that GPAM knockdown attenuated the effect of miR-328 knockdown on cell proliferation at 48 and 72 h, cell apoptosis rate, and cell invasion in both HuCCT1 and TFK1 cells (all *P <*0.05) (**Figures 7A–E**).

### Lnc-PKD2-2-3/miR-328/GPAM Network Regulated CCA Sensitivity to 5-FU

**Figure 8:**
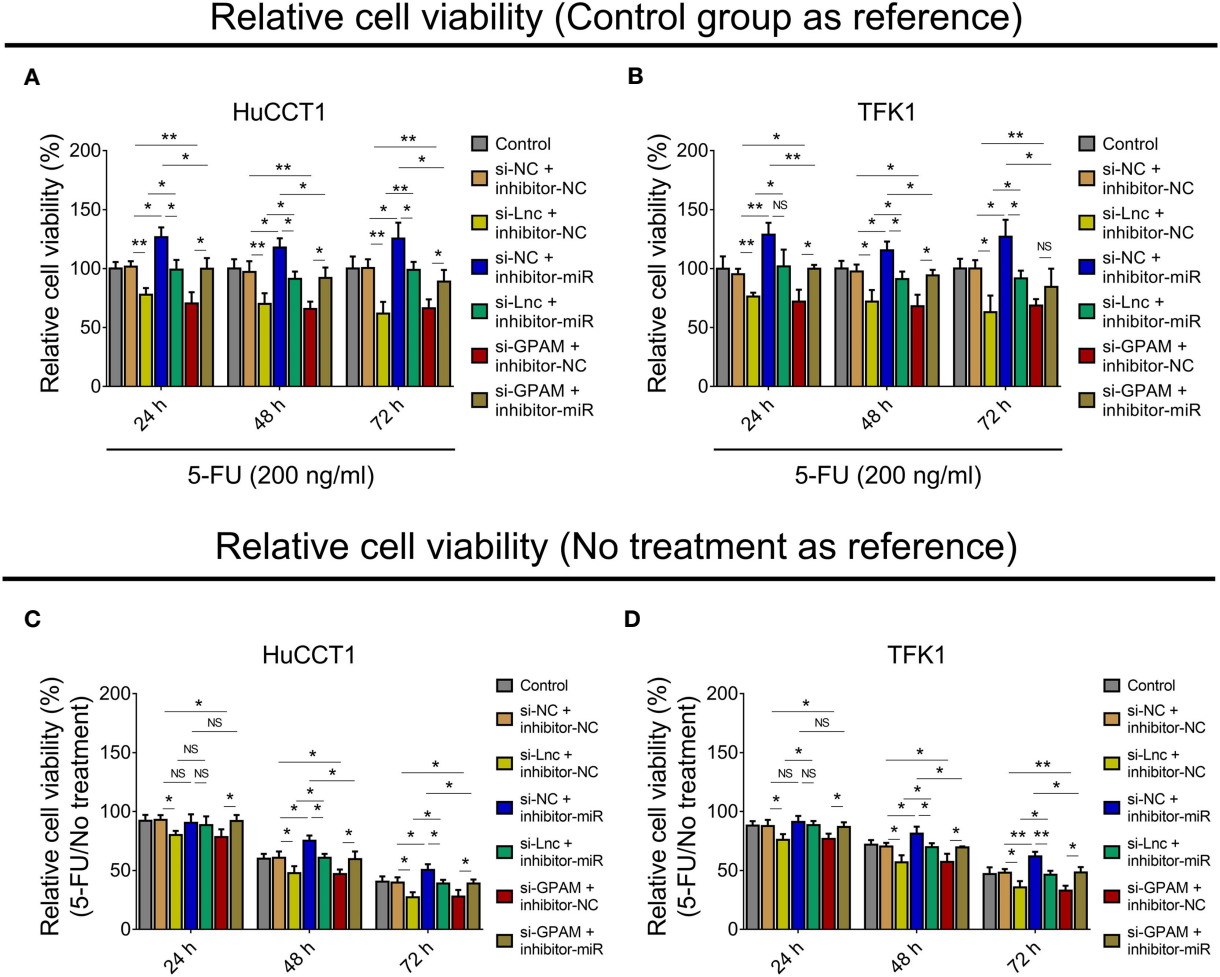
Effect of lnc-PKD2-2-3/miR-328/GPAM network on 5-FU sensitivity in CCA cell lines. Relative cell viability (when setting control group as reference) among groups after transfection at 24, 48, and 72 h in HuCCT1 cells **(A)** and TFK1 cells **(B)**; Relative cell viability (5-FU/No treatment) (when setting no treatment as reference) among groups after transfection at 24, 48, and 72 h in HuCCT1 cells **(C)** and TFK1 cells **(D)**. NS, not significant; *P <0.05; **P <0.01.

Since our previous study observed that lnc-PKD2-2-3 modified CCA sensitivity to 5-FU (19), we further explored whether the lnc-PKD2-2-3/miR-328/GPAM network regulated this issue. Then, relative cell viability when setting the control group as reference was calculated, which disclosed that relative cell viability was reduced by lnc-PKD2-2-3 knockdown and GPAM knockdown, while it was enhanced by miR-328 knockdown in both HuCCT1 and TFK1 cells (all *P <*0.05) (**Figures  8A, B**). Furthermore, miR-328 knockdown weakened the effect of lnc-PKD2-2-3 knockdown, and the effect of GPAM knockdown retarded miR-328 knockdown on the relative cell viability of HuCCT1 and TFK1 cells.

Subsequently, the relative cell viability when setting no treatment as reference (5-FU/No treatment) was also calculated, and it was observed that relative cell viability (5-FU/No treatment) was reduced by lnc-PKD2-2-3 knockdown at 24, 48, and 72 h, and decreased by GPAM knockdown at 24, 48, and 72 h, while it was elevated by miR-328 knockdown at 48 and 72 h (all *P <*0.05) in HuCCT1 cells. Meanwhile, miR-328 knockdown impaired the effect of lnc-PKD2-2-3 knockdown, GPAM knockdown attenuated the effect of miR-328 knockdown on the relative cell viability (5-FU/No treatment) of HuCCT1 cells (**Figure 8C**). Meanwhile, the effects of lnc-PKD2-2-3 knockdown, miR-328 knockdown, and GPAM knockdown on the relative cell viability (5-FU/No treatment) of TFK1 cells showed similar trends as those of HuCCT1 cells (**Figure 8D**).

### *In Vivo* Validation

**Figure 9:**
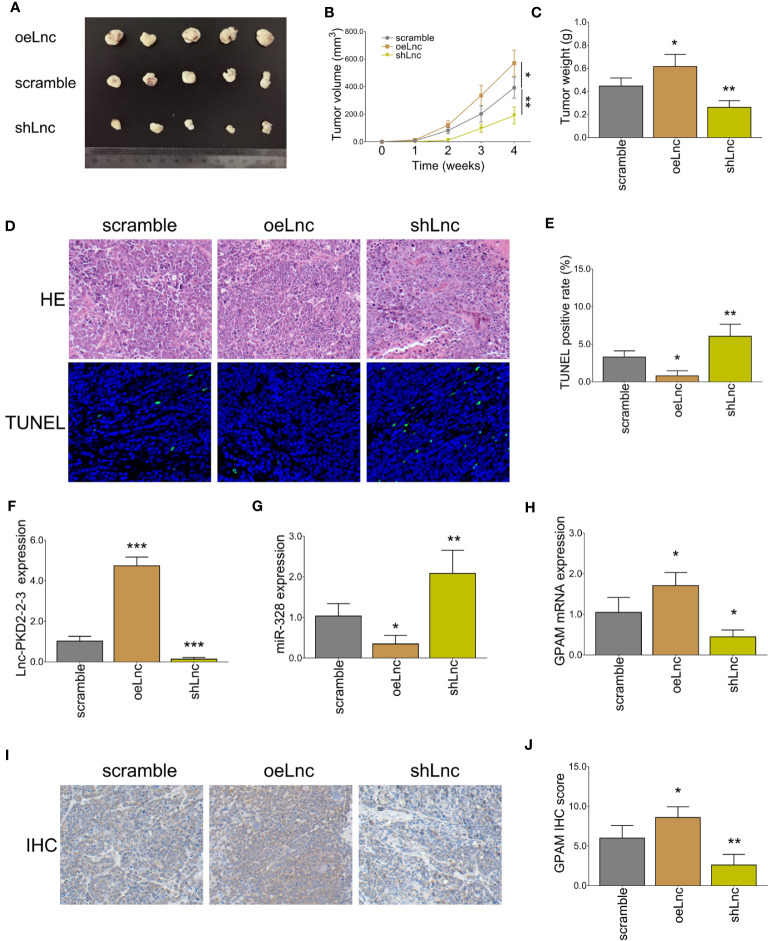
*In vivo* xenograft mice experiments. Tumor volume images **(A)**, tumor volume calculation **(B)**, tumor weight **(C)**, HE and TUNEL staining examples **(D)**, tumor apoptosis rate **(E)**, lnc-PKD2-2-3 expression **(F)**, miR-328 expression **(G)**, GPAM mRNA expression **(H)**, GPAM IHC staining examples **(I)**, and GPAM IHC score calculation **(J)** among three groups. *P <0.05; **P <0.01; ***P <0.001.

*In vivo* experiments revealed that lnc-PKD2-2-3 overexpression increased tumor volume and weight (both *P <*0.05), whereas lnc-PKD2-2-3 knockdown decreased tumor volume and weight (both *P <*0.01) in xenograft mice (**Figures 9A–C**). HE staining revealed that the stenosis area was attenuated by lnc-PKD2-2-3 overexpression (**Figure 9D**). TUNEL staining showed tumor apoptosis was reduced by lnc-PKD2-2-3 overexpression (*P <*0.05), but was enhanced by lnc-PKD2-2-3 knockdown (*P <*0.01) (**Figures 9D,E**). Additionally, lnc-PKD2-2-3 and GPAM was promoted, miR-328 was repressed by lnc-PKD2-2-3 overexpression (all *P <*0.05); meanwhile, lnc-PKD2-2-3 knockdown exhibited the opposite effect on their expression (all *P <*0.05) (**Figures 9F–J**).

## Discussion

Benefiting from the advancement of high-throughput sequencing and microarray technology, tens of thousands of lncRNAs have been discovered (20–23). Most of their functions are obscure since they lack direct protein coding ability and have just been introduced for less than twenty years. In respect of CCA, some interesting studies have uncovered a few functional lncRNAs involved in its pathogenesis, treatment sensitivity, and even serve as diagnostic/prognostic biomarkers. To give examples, a study observed that lncRNA SNHG12 enhances CCA growth and metastasis by regulating miR-199a mediated Klotho, which shows potency as a treatment target (24); another study revealed that lncRNA CASC2 targets miR-18a to increase SOCS5 and suppress CCA metastasis and epithelial–mesenchymal transition (EMT) (25). Besides, a commonly investigated lncRNA, LINC00665, is reported to induce gemcitabine resistance to CCA by regulating the miR-424/BCL9L axis (26). Furthermore, a prediction model involving five lncRNAs (HULC, AL359715.5, AC006504.8, AC090114.2, and AP00943.4) shows good value for survival prognosis (27). Nevertheless, relative to the huge number of uninvestigated lncRNAs in CCA, the exploration is far from sufficient.

Based on the importance of lncRNAs engaged in CCA and its potential application for CCA treatment, our previous study applied microarray assay and RT-qPCR validation to explore the comprehensive lncRNA expression profile related to CCA (19), which found a total of 4,223 upregulated and 4,596 downregulated lncRNAs in CCA tissues than in adjacent tissues; furthermore, lnc-PKD2-2-3 not only correlated with ECOG PS, poor differentiation, and advanced TNM stage, but also linked with worse survival in CCA patients. Then, in this study, we analyzed the lncRNA and mRNA microarray data, integrated with miRNAs *via* miRanda (http://www.microrna.org/), to sort the potential lncRNA–miRNA–mRNA regulatory network implicated in CCA pathogenesis, which finally identified a possible ceRNA network (lnc-PKD2-2-3/miR-328/GPAM). Subsequently, their expressions in CCA patients were detected in the present study, which observed that lnc-PKD2-2-3 and GPAM were elevated, while miR-328 was reduced in CCA tumor tissues compared to adjacent tissues, and they were closely inter-correlated with each other. The possible explanations are: (1) lnc-PKD2-2-3 and GPAM reflect elevated ability of proliferation of cells; meanwhile, CCA tumor tissues present with high proliferation ability, therefore they were increased in CCA tumor tissues (19, 28). (2) miR-328 inhibits tumor growth, which decreases the risk of the formation of CCA (29, 30). (3) lnc-PKD2-2-3 direct binds to miR-328 to silence its expression; meanwhile, miR-328 direct binds to GPAM to silence its expression. These are validated in our subsequent experiments; therefore, they are inter-correlated with each other in CCA tissues. Furthermore, lnc-PKD2-2-3, miR-328, and GPAM were also observed to be linked with advanced tumor features such as differentiation and tumor stage in CCA patients, which result from regulation of tumor stemness, growth, and metastasis (19, 28–30). A point could be noticed in our study: after transfection, western blot and RT-qPCR were performed to check the transfected efficiency at 24 and 48 h, while the transfected efficiency was higher at 48 h compared to 24 h, so only the related data was presented (48 h), and the following experiments were carried out based on 48 h after transfection.

Inspired by our previous study on the regulation of lnc-PKD2-2-3 on CCA stemness and drug resistance (19), as well as the above-mentioned clinical data observed in our study, we explored the lnc-PKD2-2-3 on CCA cellular functions. It was discovered that lnc-PKD2-2-3 was increased in most CCA cell lines than in the control cell line, and it promoted CCA cell proliferation and invasion but repressed cell apoptosis. The possible explanations are (1) lnc-PKD2-2-3 upregulate CD133 and OCT4, to increase the cell growth and invasion in CCA (19); (2) lnc-PKD2-2-3 targets anti-oncogene miR-328 to realize the CCA proliferation and invasion (31); (3) lnc-PKD2-2-3 may show similar oncogene role as its parent gene PKD2 does, to promote CCA proliferation and invasion (32, 33).

To further identify the deep intercorrelation of lnc-PKD2-2-3 with miR-328 and GPAM at the molecule level, subsequent rescue experiments and luciferase gene reporter assays were performed in our present study. Then it was observed that lnc-PKD2-2-3 promoted CCA proliferation and invasion by targeting miR-328; miR-328 regulated CCA proliferation and invasion by targeting GPAM; furthermore, lnc-PKD2-2-3 directly bound miR-328 and miR-328 directly bound GPAM. These suggest the ceRNA network of lnc-PKD2-2-3/miR-328/GPAM is closely engaged in CCA progression and 5-FU sensitivity. The possible reasons derived are from (1) the anti-oncogene role of miR-328 *via* multiple pathways (such as PI3K/AKT, MMP16, Notch) in cancers (34–36), while some previous studies also show contradictory data that miR-328 acts as an oncogene in some cancers (37, 38). (2) The carcinogenic role of GPAM in cancers, while related data are not sufficient, only a few studies are identified (28, 39, 40). (3) The sequence natures of lnc-PKD2-2-3, miR-328, and GPAM contribute to their ceRNA network.

Furthermore, to validate the role of lnc-PKD2-2-3 in regulating CCA progression, xenograft mouse experiments were performed. Then it was observed that lnc-PKD2-2-3 overexpression promoted tumor volume and weight but repressed tumor apoptosis in xenograft mice; meanwhile, it increased GPAM expression but decreased miR-328 expression. Conversely, lnc-PKD2-2-3 knockdown exhibited the opposite effects. These data further confirmed our findings.

In conclusion, the lnc-PKD2-2-3/miR-328/GPAM ceRNA network promotes CCA proliferation, invasion, and chemoresistance, which could serve as a treatment target for CCA.

## Data Availability Statement

The original contributions presented in the study are included in the article/**Supplementary Material**. Further inquiries can be directed to the corresponding author.

## Ethics Statement

The studies involving human participants were reviewed and approved by The 2nd Affiliated Hospital of Harbin Medical University. The patients/participants provided their written informed consent to participate in this study.

## Author Contributions

ZZ conceived and designed the study. LZ and DM collected and analyzed the data. FL, GQ, and DS prepared the figures and tables. LZ, DM, FL, GQ, and DS wrote the manuscript. ZZ revised the manuscript. All authors contributed to the article and approved the submitted version.

## Conflict of Interest

The authors declare that the research was conducted in the absence of any commercial or financial relationships that could be construed as a potential conflict of interest.

## Publisher’s Note

All claims expressed in this article are solely those of the authors and do not necessarily represent those of their affiliated organizations, or those of the publisher, the editors and the reviewers. Any product that may be evaluated in this article, or claim that may be made by its manufacturer, is not guaranteed or endorsed by the publisher.

## Supplementary Material

Supplementary Figure 1 Luciferase reporter gene assay in CCA cell lines. Relative activity of luciferase gene reporter assay of lnc-PKD2-2-3 and miR-328 in HuCCT1 cells **(A)** and TFK1 cells **(B)**. Relative activity of luciferase gene reporter assay of miR-328 and GPAM in HuCCT1 cells **(C)** and TFK1 cells **(D)**. NS, not significant; ** *P*<0.01.
