# Supplementary figures and images for: Lnc-PKD2-2-3/miR-328/GPAM ceRNA Network Induces Cholangiocarcinoma Proliferation, Invasion and 5-FU Chemoresistance
Source: Front Oncol. 2022 Jul 29;12:871281. doi: 10.3389/fonc.2022.871281 (PMC9372454; doi:10.3389/fonc.2022.871281)

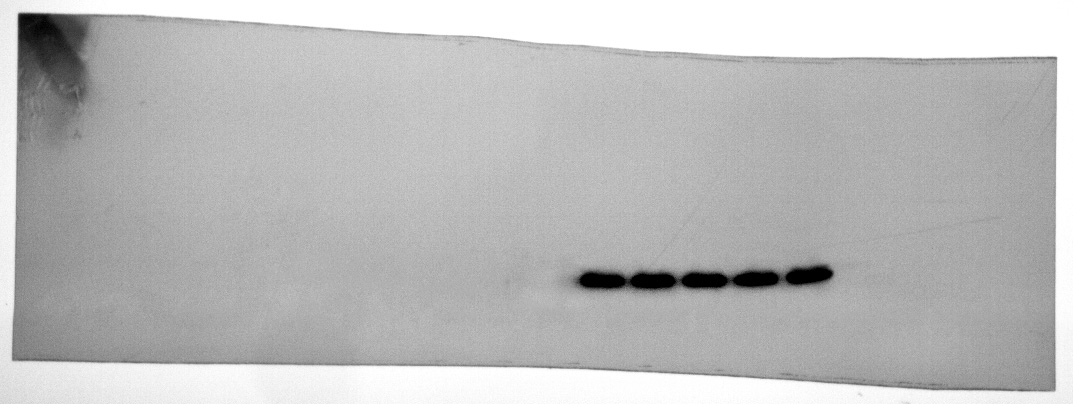

Supplement: Supplementary Figure 1 — Luciferase reporter gene assay in CCA cell lines. Relative activity of luciferase gene reporter assay of lnc-PKD2-2-3 and miR-328 in HuCCT1 cells (A) and TFK1 cells (B). Relative activity of luciferase gene reporter assay of miR-328 and GPAM in HuCCT1 cells (C) and TFK1 cells (D). NS, not significant; ** P<0.01. [file Image_1.jpeg]

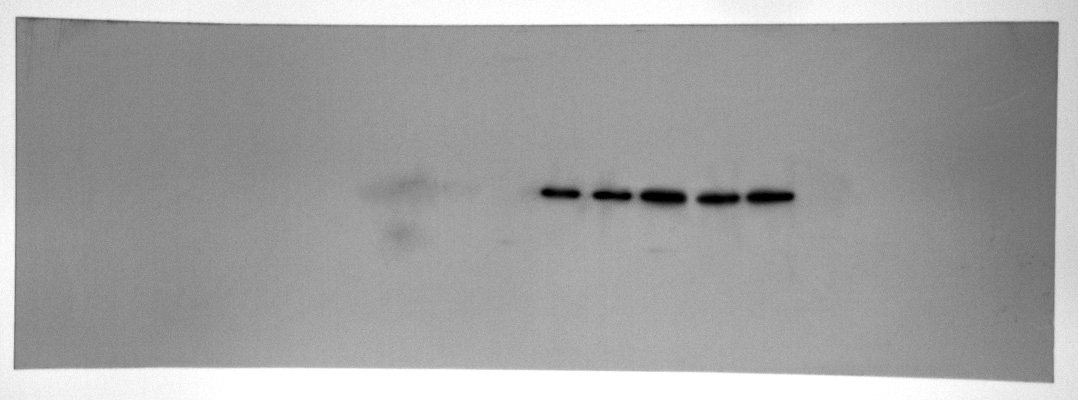

Supplement: Supplementary file 2 [file Image_2.jpeg]

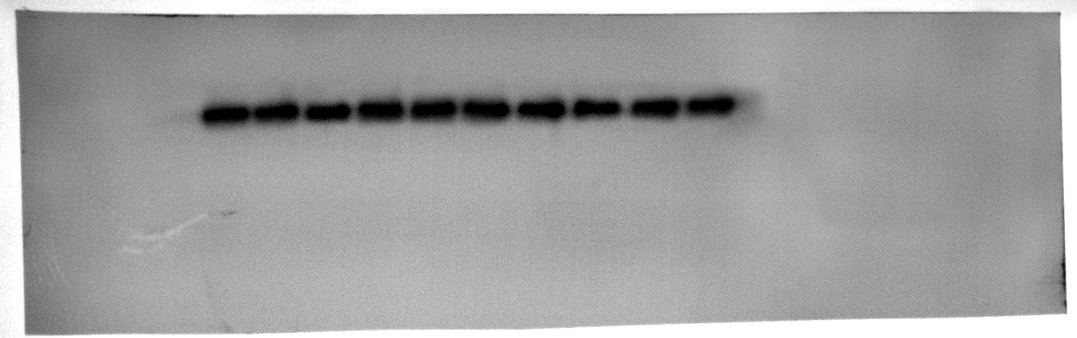

Supplement: Supplementary file 3 [file Image_3.jpeg]

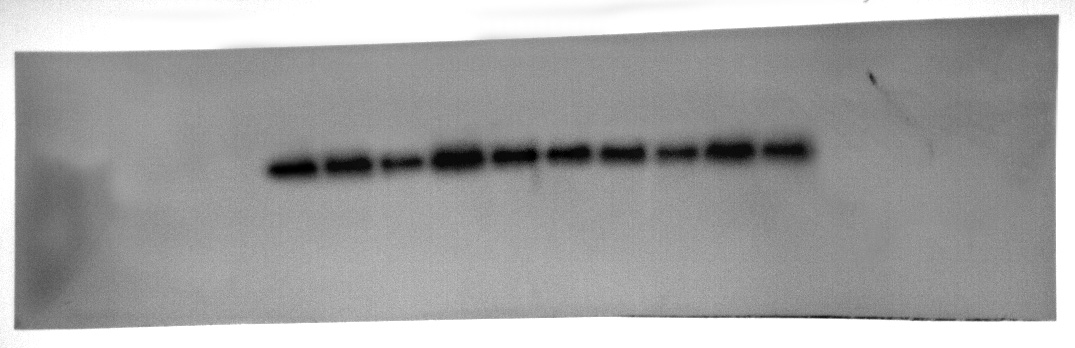

Supplement: Supplementary file 4 [file Image_4.jpeg]

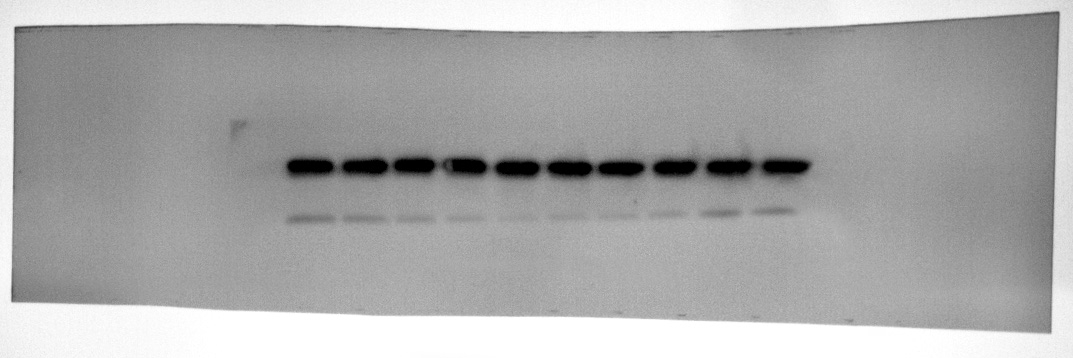

Supplement: Supplementary file 5 [file Image_5.jpeg]

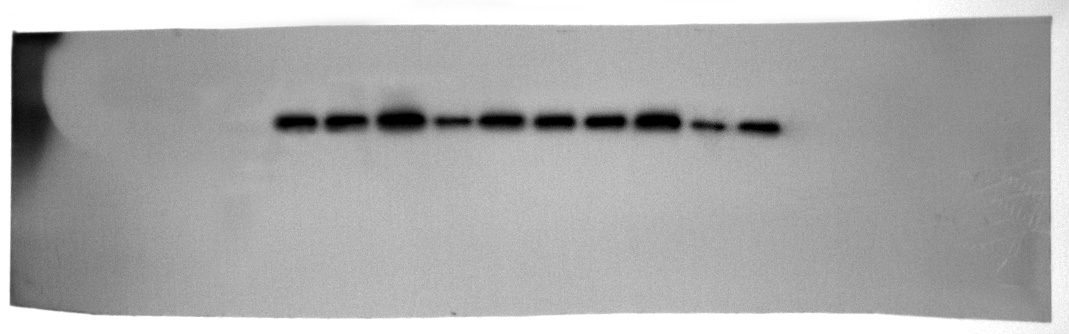

Supplement: Supplementary file 6 [file Image_6.jpeg]

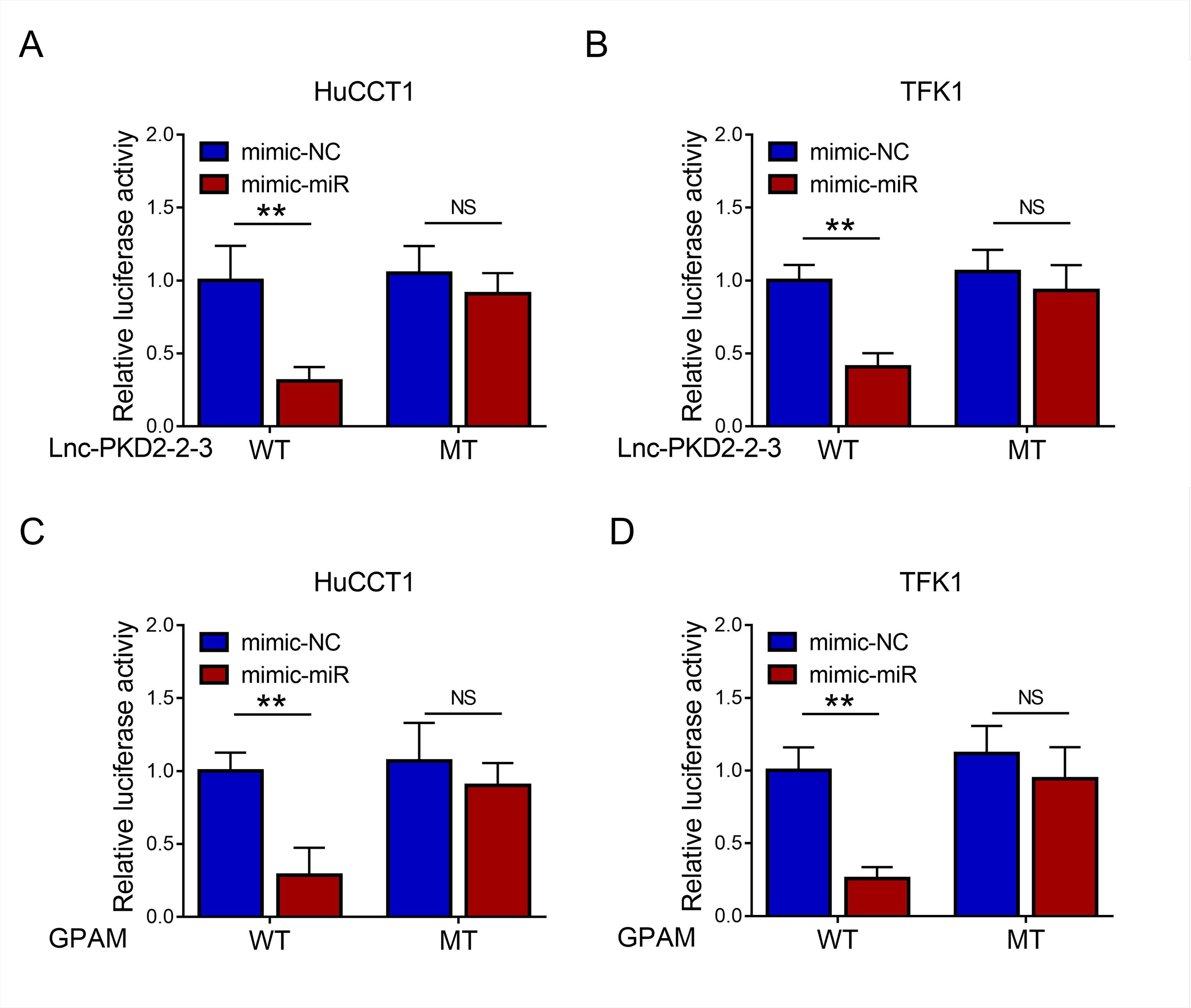

Supplement: Supplementary file 7 [file Image_7.tif]
